# MORDOR II: Persistence of Benefit of Azithromycin for Childhood Mortality

**DOI:** 10.1056/NEJMoa1817213

**Published:** 2019-06-06

**Authors:** Jeremy D. Keenan, Ahmed M. Arzika, Ramatou Maliki, Nameywa Boubacar, Sanoussi Elh Adamou, Maria Moussa Ali, Catherine Cook, Elodie Lebas, Ying Lin, Kathryn J. Ray, Kieran S. O’Brien, Thuy Doan, Catherine E. Oldenburg, E. Kelly Callahan, Paul M. Emerson, Travis C. Porco, Thomas M. Lietman

**Affiliations:** Francis I Proctor Foundation, UCSF; Department of Ophthalmology, UCSF; The Carter Center; The Carter Center; The Carter Center; The Carter Center; The Carter Center; Francis I Proctor Foundation, UCSF; Francis I Proctor Foundation, UCSF; Francis I Proctor Foundation, UCSF; Francis I Proctor Foundation, UCSF; Department of Epidemiology and Biostatistics, UCSF; Francis I Proctor Foundation, UCSF; The University of California, Berkeley School of Public Health; Francis I Proctor Foundation, UCSF; Department of Ophthalmology, UCSF; Francis I Proctor Foundation, UCSF; Department of Ophthalmology, UCSF; Department of Epidemiology and Biostatistics, UCSF; Institute for Global Health Sciences, UCSF; The Carter Center; The International Trachoma Initiative; Emory University; Francis I Proctor Foundation, UCSF; Department of Ophthalmology, UCSF; Department of Epidemiology and Biostatistics, UCSF; Francis I Proctor Foundation, UCSF; Department of Ophthalmology, UCSF; Department of Epidemiology and Biostatistics, UCSF; Institute for Global Health Sciences, UCSF

## Abstract

**Background.** MORDOR I found that 2 years of biannual mass azithromycin administration reduced post-neonatal childhood mortality by 18% in Niger. Over time, this benefit could increase with each distribution or wane due to antibiotic resistance. Here in MORDOR II, we treated communities in both arms for an additional year with azithromycin, resulting in a randomized comparison of the first versus the third year of treatment.

**Methods.** MORDOR I-Niger originally randomized 594 communities to 4 biannual distributions of either azithromycin or placebo to children aged 1-59 months. In MORDOR II, all communities received 2 additional biannual azithromycin distributions. All-cause mortality was assessed during a biannual census by enumerators masked to original assignment.

**Results.** Mean azithromycin coverage was 91.3% (SD ±7.2%) in the communities receiving the first year and 92.0% (±6.6%) in those receiving the third year of azithromycin. Mortality was 24.0 per 1,000 person-years (95% CI, 22.1—26.3) in communities randomized to the first year, and 23.3 per 1,000 person-years (95% CI, 21.4—25.5) in those randomized to the third year of treatment, with no significant difference between arms (*p*=0.55). In communities originally receiving placebo, mortality decreased 13.3% (95% CI, 5.8%—20.2%, *p*=0.007) when treated with azithromycin. In communities continuing to receive azithromycin, the mortality reduction was not significantly different in the third year (-3.6%, 95% CI, -12.3%—4.5%, *p*=0.50).

**Conclusions.** We found no evidence that the effect of mass azithromycin on childhood mortality waned in the third year of treatment. Childhood mortality fell significantly when placebo-treated communities were provided azithromycin.

## Introduction

MORDOR I (*Macrolides Oraux pour Réduire les Décès avec un Oeil sur la Résistance*) found that biannual azithromycin distributions reduced childhood mortality by 14% in communities in Niger, Malawi, and Tanzania. The greatest observed benefit was seen in Niger, with 18% fewer deaths in communities randomized to azithromycin compared to those randomized to placebo. This observed effect could decrease or increase over time for a number of reasons. For example, a beneficial effect of azithromycin might wane with selection of antibiotic-resistant bacteria. This is certainly possible, since mass azithromycin distributions in trachoma programs have selected for macrolide-resistant strains of *Streptococcus pneumoniae and Escherichia coli*, and since azithromycin clearly selected for resistance in Niger during MORDOR I.^1-9^ Or azithromycin might delay the death of a frail child, but not ultimately prevent it. Such an effect could occur if antibiotic distributions diminished the development of protective immunity in a population by reducing its exposure to pathogens. On the other hand, the observed efficacy actually increased with each distribution during the 2 years of MORDOR I, suggesting the possibility of an enhanced effect with additional treatment.^10^ Such an effect could be explained by cumulative reduction in pathogens with each distribution, or if resistant bacteria were less fit. Moreover, efficacy could improve over time due to better implementation with experience.

Here in MORDOR II, we provided biannual azithromycin to both the original placebo-treated and azithromycin-treated arms in the Nigerien communities of MORDOR I for an additional year. This resulted in a randomized comparison of the first year to the third year of mass azithromycin treatment. As azithromycin affects transmissible diseases, treating an individual may influence others in the same community. Thus randomization and intervention were at the community level, and inference of efficacy was made at the community level. MORDOR II continued the large simple trial paradigm of MORDOR I, with a straightforward intervention and primary outcome.^11^

## Methods

### Eligibility

This continuation study was planned only in the Niger districts of MORDOR I, not in the lower mortality sites of Malawi or Tanzania. The Niger component of MORDOR I was conducted in the departments of Boboye and Loga. The randomization unit was the *grappe*, and those with a population between 200 and 2,000 inhabitants on the most recent pre-MORDOR I census were eligible for enrollment. Communities remained in the continuation study MORDOR II even if the population had drifted out of this range. All children aged 1-59 months (truncated to month) and weighing at least 3,800 grams were eligible for treatment.

#### Radomizataion and masking

The original MORDOR I randomization and interventions were performed at the community level. The randomization list was generated by TCP using R (R Foundation for Statistical Computing, Vienna, Austria) and implemented by TCP, KJR, and necessary members of the Pfizer team. While all communities received azithromycin in MORDOR II, participants and observers remained masked to the original treatment arm from MORDOR I.

#### Census

A house-to-house census was performed during the 2 additional 6-month periods in the same manner as in MORDOR I.^10^ All households in the community were entered into a custom- built mobile application (Conexus Inc., Los Gatos, CA), with the head of household and the GPS coordinates facilitating identification of the household at the subsequent census. All children in the household aged 1-59 months were enumerated. The vital status (alive, dead, or unknown) and residence (moved within community, moved outside community, or unknown) were recorded for each child. The vital status of children enrolled in the preceding census who had aged past 59 months was also assessed, although these children were not included in the next study period. Pregnant women and children under the age of 1 month were recorded in the application in anticipation of enrollment in the subsequent census. Communities were censused in the same general order each period. Data were uploaded to the Salesforce Cloud Database Service (Salesforce.com, San Francisco, CA), and data cleaning was performed in Salesforce.com, Stata (Statacorp, College Station, TX), and R.

### Intervention

Each child aged 1-59 months at the census was offered a single, directly observed dose of oral azithromycin (Pfizer, Inc., New York, NY). A volume of suspension corresponding to at least 20 mg/kg was given by height-stick approximation according to Niger’s trachoma program guidelines, or by weight for those children unable to stand (typically those under 1 year of age). No azithromycin tablets were used, only suspension, and children known to be allergic to macrolides were not treated. Treatment was administered at the census and during additional visits in an attempt to achieve at least 80% coverage. Administration of study medication was documented for each child in the mobile application, with community coverage calculated relative to the census data. Serious adverse events other than death within 2 weeks of the outcome were reported to study personnel.

### Primary outcome

The pre-specified primary outcome was the community-level, all-cause mortality rate determined by biannual census. Each inter-census period was treated separately, with a mortality event counting only when a child was recorded as being alive and living in the household at one census, and recorded as having died while residing in the community at the subsequent census. By design, no attempt was made to track down the status of a child after they had moved outside the community. Person-time at risk was calculated as days between consecutive censuses, with children who moved, died, or had an unknown follow-up status contributing half the inter-census period. All children documented as alive and present in the household at the initial census of each inter-census period were included in the analysis. No changes to trial methods or outcomes were made after the continuation trial had commenced.

### Sample size and statistical analysis plan

This continuation study was pre-specified before the initiation of the original MORDOR I trial, contingent on finding a significant beneficial effect. The original sample size for MORDOR-I Niger estimated that 624 clusters would provide 80% power to detect a 15% reduction in mortality, assuming a community size of 668, 17% of the population in the target range of 1-59 months, a death rate of 2% per year, a coefficient of variation (CV) of 0.51, and loss to follow-up of 10% per year (Statistical Analysis Plan). Updating calculations based on results from MORDOR I-Niger, using the observed CV of 0.34 and a death rate of 2.5% per year, resulted in 80% power to detect a 15.5% effect size for MORDOR II. Because all communities were treated biannually, no interim efficacy or futility stopping rule was included, although a Data and Safety Monitoring Committee of 3 individuals reviewed the data on the completion of the year (Supplemental Material). For the randomized comparison of communities receiving their first year of azithromycin distributions versus those receiving their third year of antibiotics, the pre-specified primary analysis was negative binomial regression of the number of deaths per community, with treatment arm as a predictor and person- time at risk as an offset. Hypothesis testing was 2-sided, with an alpha of 0.05. A P-value was determined by Monte Carlo permutation testing (10,000 replications). Intra-cluster correlations were accounted for by using community-level data and community-level heterogeneity taken into account by the dispersion parameter in the negative binomial regression. 

### Secondary outcomes

Mortality was compared longitudinally within each arm using similar methods, contrasting the first 2 years of treatment (placebo or azithromycin) with the third year of treatment (azithromycin in both arms), clustering on community. All statistical analyses were conducted in R.

### Ethics

Approval for the study was obtained from the ethical committees of the Niger Ministry of Health, the UCSF Institutional Review Board, and Emory University. Informed consent was obtained from the local Ministry of Health, village leaders, and guardians of children. No incentives were offered for participation. The study was undertaken in accordance with the Declaration of Helsinki. The study was designed by authors JDK, AMA, PME, TCP, and TML, data gathered by JDK, AMA, RM, NB, SEA, MMA, CC, EL, KSO, TD, CEO, EKC, and TML, and data analyzed by JDK, YL, KJR, TCP, and TML. The initial draft was written by TML, with all coauthors participating in editing and agreeing to publication.

## Results

### Participant flow

**Figure 1:**
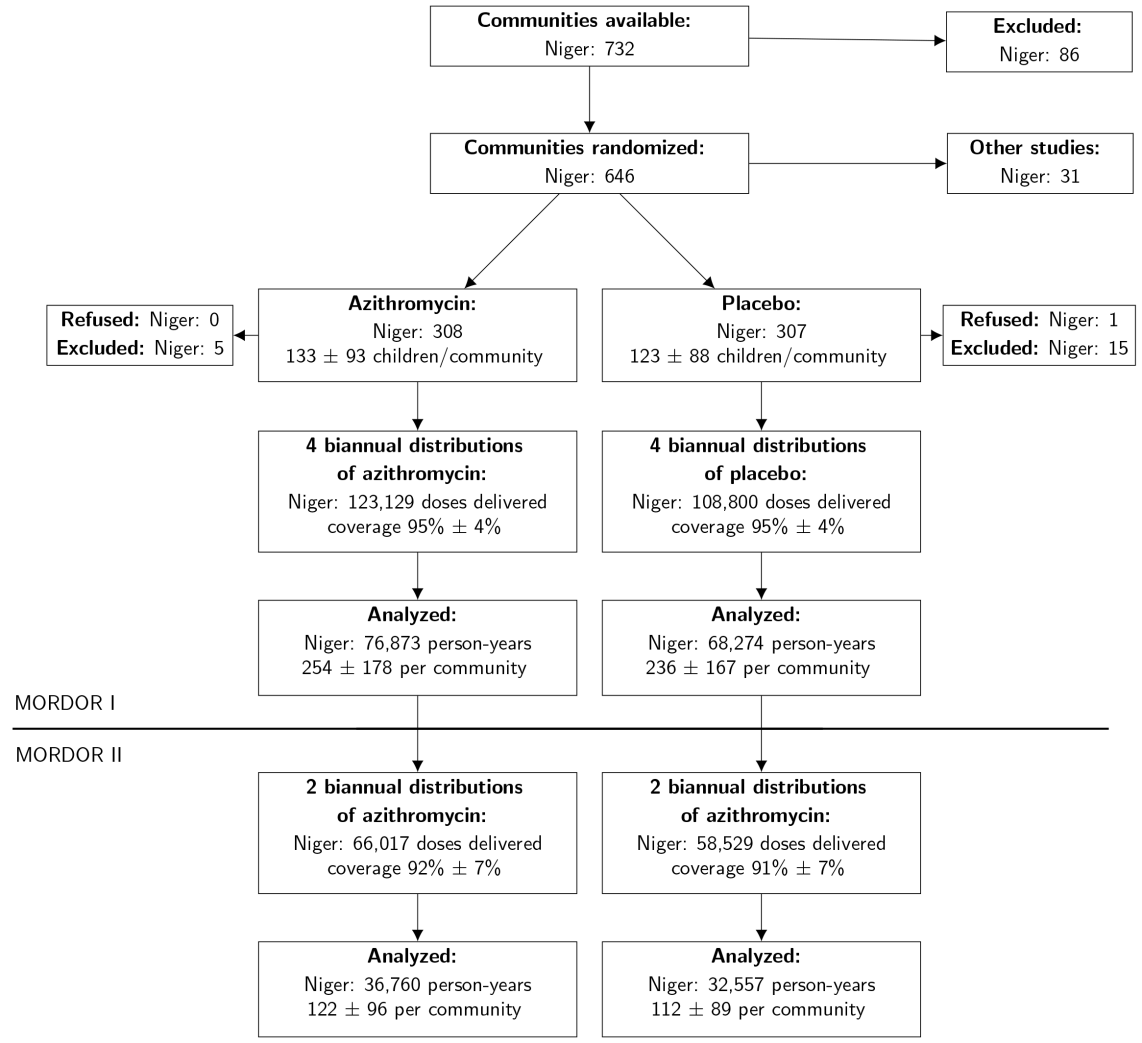
In MORDOR I, communities were enrolled and randomly assigned to 4 biannual distributions of azithromycin or placebo. In MORDOR II, these same communities were followed, with both arms offered 2 biannual distributions of azithromycin. Distribution by randomization unit is expressed as the estimated mean (±SD) for the population. No communities were lost to follow-up during MORDOR I or MORDOR II.

As displayed in Figure 1, all 594 communities from MORDOR I were followed in MORDOR II. No communities were lost to follow-up. Census periods were from February 2017 to August 2017, September 2017 to January 2018, and Feburary 2018 to August 2018. Demographic characteristics of communities in both arms at 24 months are displayed in Table 1.

**Table 1.** Demographic characteristics of communities and participants at the start of MORDOR II

Characteristic	Treatment Arm	
	Placebo	Azithromycin
Communities (number)	291	303
Children 1-59 months (number)	33,294	37,497
Children per community (mean ± sd)	114±80	124±86
Male sex	51.3%	51.2%
Age group	
1-5 mo	5.9%	6.3%
6-11 mo	9.6%	9.5%
12-23 mo	23.1%	23.0%
24-59 mo	61.4%	61.2%

In MORDOR II, azithromycin coverage averaged 92.0% (standard deviation ±6.6%) in the original azithromycin-treated communities and 91.3% (±7.2%) in the original placebo-treated communities (Supplementary Table 1). The census status was recorded as moved or unknown in 4079 of 64,225 cases (6.4%) in those communities receiving their first year of azithromycin, and as moved or unknown in 4685 of 72,108 cases (6.5%) in those communities receiving their third year of azithromycin, with no significant difference between arms (*p*=0.48, Supplementary Table 2).

**Table 2.** Childhood mortality rate over time

	Distribution year	Mortality Rate in children 1-59 months Deaths per 1000 person-years (95% CI)		Difference between arms (randomized comparison)
		Placebo	Azithromycin	
**MORDOR I**	**1**	26.3 (24.2—28.8)	21.9 (20.2—24.2)	16.0% (5.7%—25.1%) *P=0.003*
	**2**	28.0 (25.8—30.5)	22.4 (20.6—24.3)	20.3% (10.6%—28.9%) *P=0.0001*
		**Azithromycin**	**Azithromycin**	
**MORDOR II**	**3**	24.0 (22.1—26.3)	23.3 (21.4—25.5)	3.5% (-8.3%—14%) *P=0.55*
	**Difference between MORDOR I and II (longitudinal comparison)**	13.3% (5.8%—20.2%) *P=0.007*	-3.6% (-12.3%—4.5%) *P=0.50*	

### Primary outcome

**Figure 2:**
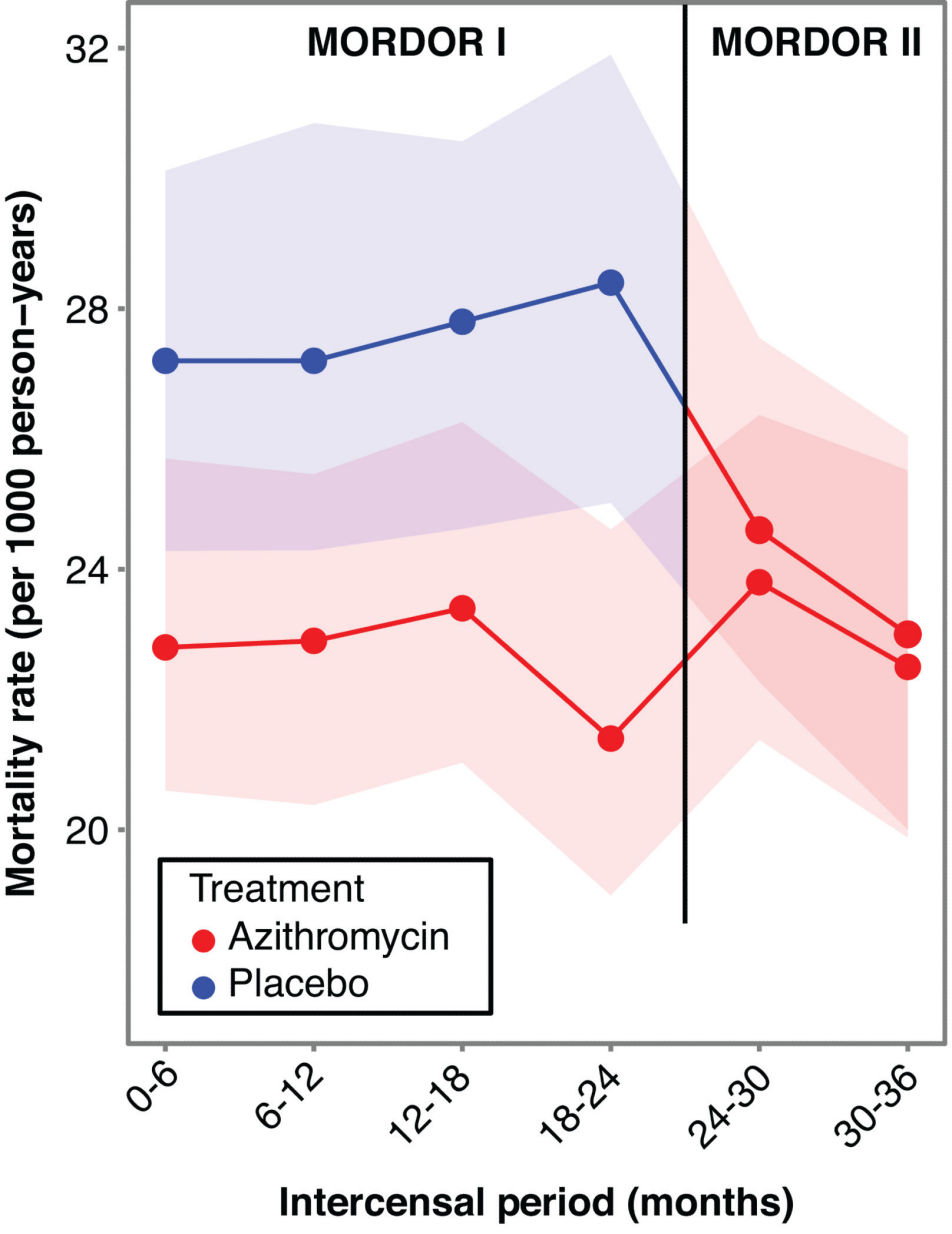
All-cause mortality rate in 1-59 month old children over time in communities randomized to 2 years of treatment with biannual placebo and the third year with biannual azithromycin (blue, with 95% CI in lighter blue), and in communities randomized to 3 years of biannual azithromycin (red, with 95% CI in lighter red). In MORDOR II, we were unable to show a statistically signficant difference between the 2 arms in year 3 (*p*=0.55). Mortality did decrease significantly in the originally placebo-treated communities (-13.0%, 95% CI, -21.5% to -3.7%, *p*=0.008). In the communities originally receiving azithromycin, mortality was not significantly different in a third year of azithromycin (2.1%, 95% CI, -7.6 to 12.6%, *p*=0.69). Note that the annual mortality rates used in this study are expected to be several fold lower than the Under 5 Mortality Rate (U5MR), which is the number of live births that do not survive till their fifth birthday.

Mortality rates by treatment arm are displayed by inter-census period (Figure 2) and by year (Table 2). Communities randomized to the first year of azithromycin distribution experienced 24.0 deaths per 1,000 person-years (95% CI, 22.1 to 26.3), and those randomized to the third year of azithromycin distribution 23.3 deaths (95% CI, 21.4 to 25.5) per 1,000 person- years. We found no evidence that the first year of treatment had a greater effect than the third year of treatment, with a relative 3.5% (95% CI -8.3% to 14%) more deaths in those communities randomized to the first year of treatment (*p*=0.55, Table 2). 

### Secondary outcomes

In the communities originally receiving 2 years of biannual placebo distribution, mortality decreased (-13.3%, 95% CI, 5.8% to -20.2%, *p*=0.007) over the next year when treated with biannual azithromycin. In the communities originally receiving azithromycin, the mortality reduction was not significantly different in the third year compared to the first 2 years (-3.6%, 95% CI, -12.3% to 4.5%, *p*=0.50).

### Serious adverse events

Mortality is as reported, and medical review was unable to declare that any additional serious adverse events were possibly caused by azithromycin.

## Discussion

In MORDOR I, 2 years of biannual oral azithromycin distribution to post-neonatal preschool children significantly reduced all-cause mortality in Niger by 18%.^10^ Here in MORDOR II, both arms received an additional year of biannual azithromycin, resulting in a randomized comparison of a third year of treatment to the first year of treatment. We found no evidence that the benefit of azithromycin waned in the third year. Some had hypothesized a decrease in efficacy with more distributions due to the selection of antibiotic resistant bacteria.^12-14^ Repeated mass azithromycin distributions for trachoma have indeed selected for macrolide resistance in nasopharyngeal *S. pneumoniae* and rectal *E. coli*.^1-3,6,15^ Resistance was clearly selected for in the nasopharynx and stool in Niger in MORDOR I.^9^ Resistance emerging during mass azithromycin distributions could theoretically have curbed or even reversed any potential survival benefit. We also found no evidence that the effect of azithromycin was enhanced with additional distributions. Enhancement was possible since the overall benefit in the 3 sites of MORDOR I increased with each of the first 4 biannual distributions from 7% to 22%, although that apparent increase was not statistically significant.^10^ Here, the randomized comparison between the first and third year of treatments did not support either an increasing or decreasing effect on mortality with additional rounds distributions of azithromycin. Even longer follow-up will be necessary to determine whether the mortality effect is sustained past the third year of distributions. 

The communities receiving their first year of treatment had 13% lower mortality than they had in the previous two years of receiving placebo. While this longitudinal analysis was not a randomized comparison and is therefore subject to confounding, the result does support the original MORDOR I finding of a 14% reduction in the 3-country analysis. The mortality rate fell with the first of the two additional distributions, suggesting that cumulative treatments are not necessary to achieve efficacy. This is consistent with a secondary analysis of MORDOR I in which deaths were relatively lower in the first 3 months after a biannual distribution.^16^ The convergence of mortality rates in the two arms in MORDOR II—when both arms received the same treatment—adds some support that the difference in MORDOR I was indeed due to intervention and not from imbalanced randomization.

The study has several limitations. As a large simple trial, little information was collected on each child and community.^11^ Deaths were determined by consecutive censuses. Children who were born and died between censuses contributed neither to the death count nor person-time at risk for the primary outcome. Death rates may have differed in children who moved or had an unknown census status. While the randomized comparison assessed whether a community’s prior treatment history affected the results, it was not designed to analyze an individual’s prior treatment history. Cluster-randomized trials run the risk of contamination between arms, which could dampen the observed effect. While the intervention itself was not subject to contamination since all communities were given the same treatment, infections could spread between nearby communities and cause contamination. Although this could theoretically explain the MORDOR II findings, contamination did not prevent a highly significant result in MORDOR I, so invoking this explanation would require contamination in the third year only. No child in MORDOR had ever received azithromycin as part of a trachoma program, but macrolide use outside of the study was not recorded. As distributions were offered only biannually, a child’s first treatment might not be until 7 months of age. Supplementary treatments given during a scheduled vaccination visit to a health clinic might prove to be a more reliable way of reaching younger infants. The longitudinal comparisons of years 1 and 2 versus year 3 were not randomized. Conditions may have changed between these time periods. This study did not investigate whether morbidity increased or decreased with azithromycin. The study also did not evaluate the mechanism by which azithromycin reduced mortality, although its antimicrobial effect presumably plays a role since a majority of child deaths in this area are attributed to infectious disease.^17^ Smaller parallel trials with detailed microbiological and anthropometric assessments were conducted, and may provide insight into mechanism of action.^18, 19^ Azithromycin has been linked to cardiac death in adults, although epidemiological results are mixed and may not be relevant to children in this setting.^20-23^ Later development of atopic disease has been associated with infant antibiotic use in general, and macrolides in particular.^24^ Rare side effects, or those only apparent later in life would be difficult to assess with this study design. 

The International Trachoma Initiative has now distributed over 800 million doses of oral azithromycin in the trachoma control programs sponsored by the World Health Organization.^25^ Azithromycin has proven quite effective in reducing the prevalence of, and in some cases completely elimininating the strains of ocular chlamydia that cause the disease.^2,4,6,26-28^ The number of annual trachoma distributions is now declining as countries continue to meet control criteria.^25^ Many regions with high childhood mortality are either no longer endemic for trachoma, or never were. Thus the majority of children now being born into areas with the highest under-5 mortality will not receive azithromycin as part of trachoma programs.^29^ The treatment regimens were different for trachoma and MORDOR: annual mass azithromcyin treatment of ages 6 months and older for trachoma, and biannual distributions targeted to ages 1- 59 months in MORDOR I and II. MORDOR distributed approximately one third as many doses of azithromycin per community per year as would a trachoma program. If azithromycin for childhood mortality were targeted to areas with very high mortality such as Niger, only a fraction of the total antibiotic used in trachoma programs would be required. 

In summary, a randomized comparison found no evidence that the beneficial effect of mass biannual azithromycin distribution on childhood mortality wanes in the third year of distribution compared to the first. Biannual oral azithromycin distribution did significantly reduce mortality compared to the 2 previous years of biannual placebo distributions. This longitudinal observation supports the original MORDOR I community-randomized trial results. Selection of antibiotic resistant strains of pathogenic bacteria may eventually reduce efficacy and needs to continue to be monitored with longer follow-up.

## Supplementary appendix

Supplementary appendix
