# Supplementary material for: MORDOR II: Persistence of Benefit of Azithromycin for Childhood Mortality
Source: N Engl J Med. Author manuscript; Available in PMC 2019 Jun 6. (PMC6512890; doi:10.1056/NEJMoa1817213)
Supplement: Supplementary appendix [file NEJMoa1817213_Keenan_Supplement.pdf]

Table of Contents

Page 1: Table of Contents
Page 2: List of investigators
Page 3: Supplemental tables

MORDOR II-Niger Study Group Investigators:

University of California, San Francisco, San Francisco, CA, USA – Cindi Chen, Catherine A Cook, Sun Y Cotter, Susie Cummings, Thuy Doan, Dionna M Fry, Bruce D Gaynor, Armin Hinterwirth, Jeremy D Keenan, Elodie Lebas, Thomas M Lietman, Kieran S O'Brien, Catherine E Oldenburg, Travis C Porco, Kathryn J Ray, Philip J Rosenthal, Samarpita Sarkar, Nicole E Stoller, Benjamin Vanderschelden, John P Whitcher, Zhaoxia Zhou, Lina Zhong; *The Carter Center, Atlanta, GA, USA* – E Kelly Callahan, Aisha E Stewart; *The Carter Center Niger, Niamey, Niger* – Ahmed M Arzika, Nameywa Boubacar, Abdou Moumouni Goundara, Salissou Kane, Ramatou Maliki, Maria Moussa Ali, Sanoussi Elh Adamou; *Programme National de Santé Oculaire, Niamey, Niger* – Amza Abdou, Nassirou Beido, Boubacar Kadri; *International Trachoma Initiative, Decatur, GA, USA*- Paul M Emerson, Huub Gelderbloom, MD, PJ Hooper.

Data and Safety Monitoring Committee MORDOR I –University of Washington, Seattle, WA, USA – Judd L Walson (chair)*; Liverpool School of Tropical Medicine, Liverpool, UK* – Allen W Hightower*; Loyola University, Chicago, IL, USA* – Emily E Anderson*, Berhan Public Health & Eye Care Consultancy, Addis Ababa, Ethiopia* – Wondu Alemayehu*; Tulane University, New Orleans, LA, USA* – Latha Rajan.

Data and Safety Monitoring Committee MORDOR II – University of California Berkeley, Berkeley CA, USA– Art Reingold; University of California San Francisco, San Francisco, CA, USA – Dan Kelly (chair), George Rutherford.

Bill & Melinda Gates Foundation, Seattle, WA, USA – Rasa Izadnegahdar, Julie Jacobson, Thomas Kanyok, Erin Shutes.

Pfizer, New York, NY, USA –Julie Jensen, Chuck Knirsch, John Schenkel

Supplemental Table 1. Treatment coverage by arm and census period

Inter-census period	Mean Coverage (± SD)	
	Placebo	Azithromycin
24-30 months	91.6% (±6.2%)	91.8% (±6.1%)
30-36 months	91.0% (±8.0%)	92.1% (±7.0%)
ALL	91.3% (±7.2%)	92.0% (±6.6%)

Supplemental Table 2. Census results

Category	Treatment Arm	
	Azithromycin	Placebo
Census enrollments	72,108	64,225
Died	857	782
Moved and Unknown	4,685	4,079
Alive	66,566	59,364
